# The utility of exome sequencing for genetic diagnosis in a familial microcephaly epilepsy syndrome

**DOI:** 10.1186/1471-2377-14-22

**Published:** 2014-01-31

**Authors:** Laura M McDonell, Jodi Warman Chardon, Jeremy Schwartzentruber, Denise Foster, Chandree L Beaulieu, Jacek Majewski, Dennis E Bulman, Kym M Boycott

**Affiliations:** 1 Children’s Hospital of Eastern Ontario Research Institute, University of Ottawa, Ottawa, Ontario, Canada; 2 Department of Genetics, Children’s Hospital of Eastern Ontario, Ottawa, Ontario, Canada; 3 McGill University and Genome Quebec Innovation Centre, Montréal, Quebec, Canada; 4 Algoma Public Health, Sault Ste Marie, Ontario, Canada; 5 Department of Human Genetics, McGill University, Montréal, Quebec, Canada; 6 Department of Pediatrics, University of Ottawa, Ottawa, Ontario, Canada

**Keywords:** Primary microcephaly, Epilepsy, Whole-exome sequencing, WDR62

## Abstract

**Background:**

Despite remarkable advances in genetic testing, many adults with syndromic epilepsy remain without a molecular diagnosis. The challenge in providing genetic testing for this patient population lies in the extensive genetic heterogeneity associated with epilepsy. Even for the subset of epilepsy patients that present with a defining feature, such as microcephaly, the number of possible genes that would require interrogation by Sanger sequencing is extensive and often prohibitively expensive.

**Case presentation:**

We report a family of French Canadian descent with four adult children affected with severe intellectual disability, epilepsy and microcephaly born to consanguineous parents and evaluated by the Genetics Service to provide informed genetic counseling to unaffected family members regarding possible recurrence risks. We used whole-exome sequencing (WES) of DNA from one affected sibling as a first-line diagnostic tool and compared the prioritization of variants using two strategies: 1) focusing on genes with homozygous variants; and, 2) focusing on genes associated with microcephaly. Both approaches prioritized the same homozygous novel frameshift mutation (p.Arg608Serfs*26) in *WDR62*, a gene known to cause autosomal recessive primary microcephaly. Sanger sequencing confirmed the presence of the homozygous mutation in the other three affected siblings.

**Conclusions:**

WES and subsequent filtering of the rare variants in a single affected family member led to the rapid and cost-effective identification of a novel homozygous frameshift mutation in *WDR62,* thereby explaining the severe neurodevelopmental disorder in this family and facilitating genetic counseling. Our findings support WES as an effective first-line diagnostic tool in families presenting with rare genetically heterogeneous neurological disorders.

## Background

Although up to 70% of patients with epilepsy have an underlying genetic cause [1], most patients with epilepsy will not receive a molecular diagnosis. Genetic testing for epilepsies is challenging since genetic heterogeneity for this group of conditions is significant. For instance, epilepsy can be caused by mutations in over 260 genes and an accurate diagnosis can be particularly difficult with nonspecific epilepsy phenotypes [2]. Even when epilepsy is associated with additional features, such as microcephaly, the number of genes potentially responsible can still be significant and assessing each gene in turn by Sanger sequencing would be time-consuming and expensive. However, establishing a molecular diagnosis is important for informed genetic counseling, prenatal testing, insight into natural history and increasingly for therapeutic management as there are now many instances where medications are tailored based on molecular results [3-5].

Whole exome sequencing (WES) has emerged as a very successful tool for novel disease gene discovery [6] and its utility in the rapid and cost-effective analysis of heterogeneous disorders is being established. Both a WES approach and the application of targeted panels of channelopathy or encephalopathy genes have been successful in identifying the molecular cause for many patients with rare epilepsies [2,7]. In addition, WES may offer a rapid and less expensive first line test to establish a molecular diagnosis for disabled patients residing in remote areas where ready access to standard diagnostic assessments can be more challenging. We report here the utility of WES as the initial diagnostic tool for a consanguineous family with four affected adult children with epilepsy, microcephaly and severe intellectual disability from a remote area of Canada.

## Case presentation

### Clinical findings

**Figure 1:**
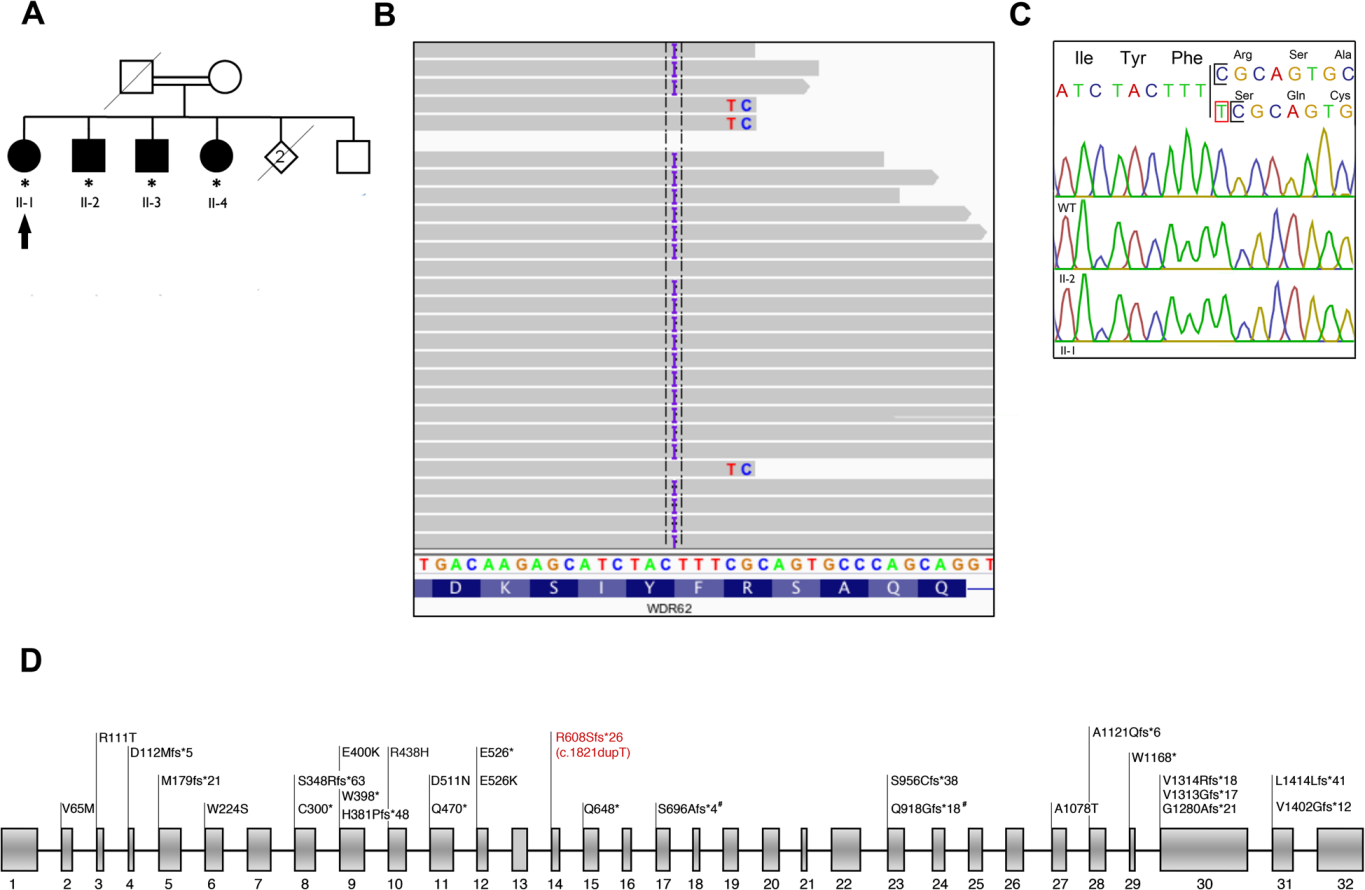
**WES identifies a novel frameshift mutation in *WDR62*. (A)** Family pedigree showing the four affected siblings (solid symbols) and unaffected family members (open symbols). Squares represent males, circles females, asterisk denote available DNA. Patient II-1 was selected for WES (black arrow). **(B)** WES in Patient II-1 revealed a homozygous single-base pair insertion (c.1821dupT) in *WDR62* producing a frameshift mutation (p.Arg608Serfs*26). **(C)** The mutation was validated by Sanger sequencing in all affected siblings (only II-1 and II-2 are shown); parental DNA was unavailable for this study. The mutation is absent in an unaffected control (WT). **(D)** Reported *WDR62* mutations in MCPH2 families. *WDR62* is located on chromosome 19q13.12 and contains 32 exons (NM_001083961.1) encoding 1523 amino acids. For clarity, reported *WDR62* mutations are annotated as peptide changes (grey). Number sign indicates the compound heterozygous mutation present in one reported family [15]. The homozygous single-base pair insertion identified in the French-Canadian family is also shown (red).

Four affected siblings (two females and two males) from a consanguineous French Canadian family presented in their 40s and 50s to a satellite genetics clinic for assessment to facilitate genetic counseling for extended family members (Figure 1A). Two developmentally normal brothers died in an accidental fire and one living brother is unaffected. The parents were from an isolated community and were distantly related. The four siblings had moderate to severe intellectual disability, epilepsy and microcephaly (Table 1). A more severe phenotype was seen in the two male siblings, including medically refractory seizures, aggression and self-injury. Due to the difficulty of performing MRI in a remote region and the long differential diagnosis, DNA was sent for WES as an initial investigation.

**Table 1:** Clinical and neuroimaging features of the four MCPH2 patients

**Patient**	**II-1**	**II-2**	**II-3**	**II-4**
Gender	F	M	M	F
Age at assessment (yrs)	59	56	55	53
**Clinical features**				
Height (cm)	158	154	166	154
Height percentile	10-25th	<5th	5th	5-10th
Occipito-frontal circumference (cm)	48.5	52	51	48
Occipito-frontal circumference (SD)	-4 to -5 SD	-2 to -3 SD	-3 SD	-4 to -5 SD
Sloping forehead	+	+	+	+
Intellectual disability	Moderate-severe	Severe	Severe	Moderate-severe
Behaviour	Pleasant	Self injurious behaviour	Self injurious behaviour	Pleasant
Tone	Normal	Hypertonia	Hypertonia	Normal
Sensory exam	Normal	Normal	Normal	Normal
Reflexes	Normal	Hyperreflexia/ankle clonus	Hyperreflexia/ankle clonus	Normal
Bladder/bowel incontinence	+/-	+/+	+/+	+/-
**Neuroimaging features**				
Microcephaly	+	Nd	Nd	+
Polymicrogyria	-	Nd	Nd	-
Cerebellar hypoplasia	-	Nd	Nd	-
Cortical thickenng	-	Nd	Nd	-
Corpus callosum hypoplasia	-	Nd	Nd	-

*Abbreviations*: *F* female, *M* male, *nd* not determined.

### Genetic studies

**Figure 2:**
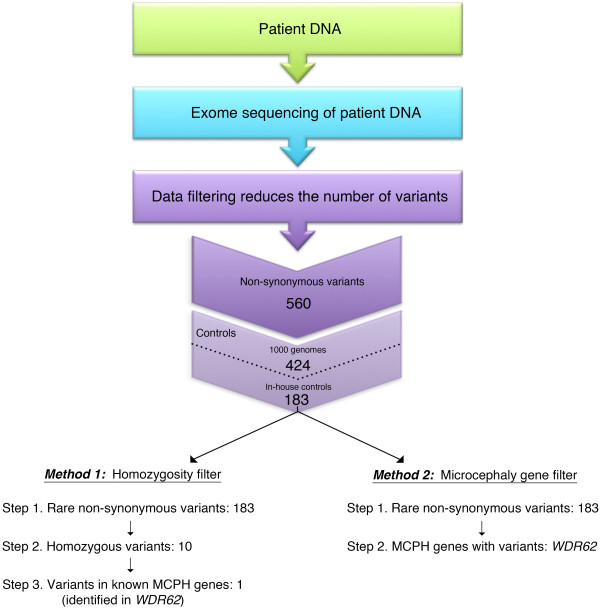
**Exome sequencing workflow.** Exome sequencing was performed on isolated DNA from Patient II-1. The data was filtered to identify non-synonymous sequence variants including frameshift and non-frameshift indels, single nucleotide variants (SNV) including stop-gain and missense SNVs, and splicing and splicing-extending variants. Variants with a frequency of less than 1% in the 1000 genomes pilot release (Nov 2010) and in 100 in-house controls were then identified. The first method of analysis focused on rare homozygous variants (Method 1) and a second, simpler approach, focused only on rare variants in disease genes known to cause microcephaly/epilepsy (Method 2). Both methods identified the homozygous mutation in *WDR62* as the cause of the neurodevelopmental disorder in this family.

Genomic DNA was extracted from whole blood obtained from the four affected individuals using the QIAamp DNA Blood Kit (Qiagen, CA, USA). DNA from Patient II-1 underwent WES. Using target capture with the Agilent SureSelect 50 Mb All Exon kit (Agilent Technologies, Santa Clara, CA) and sequencing of 100 bp paired end reads on Illumina Hiseq, we generated over 15 Gb of sequence for the sample such that approximately 90% of the coding bases of the exome defined by the consensus coding sequence (CCDS) project were covered by at least 20 reads. WES data was analyzed as previously described [8] and non-synonymous variants (frameshift and non-frameshift indels, single nucleotide variants (SNV) including stop-gain and missense SNVs, and splicing and splicing-extending variants) were identified as rare when they had a frequency of less than 1% in the 1000 genomes pilot release (Nov 2010) and in 100 in-house controls. We then compared two approaches to examine this subset of WES variants (Figure 2).

Following our preliminary filtering steps that identified 183 rare non-synonymous variants, we examined the sequencing data for homozygous variants as the pedigree was consistent with autosomal recessive inheritance and the parents were distantly related (see Method 1, Figure 2). WES identified ten exome-wide homozygous variants including a rare variant in *WDR62*, encoding a protein essential to cerebral cortex development and known to cause microcephaly [9-11] (Figure 1B) and thus felt to be a strong candidate gene for the disorder in this family. The *WDR62* mutation identified is a novel homozygous single-base pair insertion NM_001083961.1:c.1821dupT) producing a frameshift mutation (p.Arg608Serfs*26) in exon 14 (Figure 1C). The protein-truncating mutation is absent from the 6500 exomes in the NHLBI Exome Variant Server. We next examined the WES data using a second approach to identify all rare variants in genes known to be associated with epilepsy and microcephaly (see Method 2, Figure 2); the same mutation in *WDR62* was identified as the only candidate.

The mutation in *WDR62* was validated by Sanger sequencing in the other three siblings. After the diagnosis, unenhanced MRI of the brain was performed in both sisters and demonstrated microcephaly without polymicrogyria, cerebellar hypoplasia, cortical thickening or corpus callosum hypoplasia. Patient II-2 died suddenly of a cardiac arrest but was not known to have cardiac disease prior to the fatal event; the family declined an autopsy.

## Discussion

Primary autosomal recessive microcephaly (MCPH, OMIM 251200) is a genetically heterogeneous group of disorders characterized by congenital microcephaly, cognitive impairment and variable epilepsy [12]. Mutations in *WDR62* are associated with primary microcephaly-2 (MCPH2, OMIM 604317), which is believed to account for 10% of all cases of MCPH [13]. Clinical features of MCPH2 include epilepsy, marked cognitive impairment, incontinence, sloping forehead and prominent ears. Radiological features are variable and may include polymicrogyria, corpus callosum hypoplasia, heterotopias, pachygyria with cortical thickening, lissencephaly and schizencephaly, although patients can present with isolated microcephaly [14-18]. To date, 34 families have been identified with mutations in *WDR62*, the reported affected individuals ranging in age from infancy to mid-teens [14-18].We present here the oldest patients known to have *WDR62* mutations associated with MCPH, providing additional insight into the natural history and phenotypic spectrum of this disorder. It is unclear whether the sudden cardiac death for Patient II-2 is associated with *WDR62* mutations or an unrelated cardiac event. Of the mutations in *WDR62* reported thus far, approximately half have been frameshift mutations, as described here, distributed throughout the gene and predicted to result in premature truncation of the protein (Figure 1C).

At least ten genes have been associated with MCPH [*ASPM, MCPH1, WDR62, CDK5RAP2, CASC5, CENPJ, STIL, CEP135, CEP152* and *ZNF335*] and are implicated in proliferation and migration of neuronal progenitors during embryonic cortical development [19]. Clinical-radiological features have not been loci-specific and MCPH patients remain phenotypically indistinguishable [13]. Thus, conventional molecular testing can be a long and expensive process and may not yield a diagnosis. Currently, sequential analysis of the eight clinically available MCPH genes would total more than $22,000 USD. Alternatively, eight of the ten MCPH genes could be sequenced as part of a microcephaly panel for $7,000 USD (http://www.centogene.com accessed November 6^th^, 2013, eight genes). Comparatively, WES would interrogate all ten microcephaly genes and is currently available in clinical laboratories at $5000 USD per patient, making it a cost-effective solution for rapid diagnosis in the clinic.

## Conclusion

We studied four siblings from a consanguineous French Canadian family presenting with microcephaly, severe developmental delay and epilepsy. Given their relatively nonspecific phenotype which did not suggest a single likely candidate gene, sequential gene testing was not undertaken. Alternatively, WES provided a cost-effective and rapid method to identify the genetic cause of epilepsy and microcephaly in this family. We highlight, that particularly for patients in remote areas, WES via DNA blood/saliva offers a non-invasive and readily accessible method for shortening the diagnostic process for these patients.

### Consent

Informed consent was obtained for participation in the Finding of Rare Disease Genes (FORGE) Canada study. The Research Ethics Board of the Children’s Hospital of Eastern Ontario approved this study in accordance with the Declaration of Helsinki. A copy of the written consent is available for review by the Editor of this journal.

## Competing interests

The authors declare that they have no competing interests.

## Authors’ contributions

KMB and DEB directed the study. JWC, DF and KMB provided clinical data. LMM performed Sanger sequencing, genotyping studies and exome variant analysis supervised by KMB and DEB. JS and JM performed exome variant calling analysis. The manuscript was written by LMM, JWC and KMB. KMB is lead of the FORGE Canada Consortium and is assisted by CLB. All authors read and approved the final manuscript.

## Pre-publication history

The pre-publication history for this paper can be accessed here:

http://www.biomedcentral.com/1471-2377/14/22/prepub
